# Inferring the Distribution and Demography of an Invasive Species from Sighting Data: The Red Fox Incursion into Tasmania

**DOI:** 10.1371/journal.pone.0116631

**Published:** 2015-01-20

**Authors:** Peter Caley, David S. L. Ramsey, Simon C. Barry

**Affiliations:** 1 Commonwealth Scientific and Industrial Research Organization, Canberra, Australia; 2 Arthur Rylah Institute, Department of Sustainability and Environment, Victoria, Australia; Center for Cancer Research, National Cancer Institute, UNITED STATES

## Abstract

A recent study has inferred that the red fox (*Vulpes vulpes*) is now widespread in Tasmania as of 2010, based on the extraction of fox DNA from predator scats. Heuristically, this inference appears at first glance to be at odds with the lack of recent confirmed discoveries of either road-killed foxes—the last of which occurred in 2006, or hunter killed foxes—the most recent in 2001. This paper demonstrates a method to codify this heuristic analysis and produce inferences consistent with assumptions and data. It does this by formalising the analysis in a transparent and repeatable manner to make inference on the past, present and future distribution of an invasive species. It utilizes Approximate Bayesian Computation to make inferences. Importantly, the method is able to inform management of invasive species within realistic time frames, and can be applied widely. We illustrate the technique using the Tasmanian fox data. Based on the pattern of carcass discoveries of foxes in Tasmania, we infer that the population of foxes in Tasmania is most likely extinct, or restricted in distribution and demographically weak as of 2013. It is possible, though unlikely, that that population is widespread and/or demographically robust. This inference is largely at odds with the inference from the predator scat survey data. Our results suggest the chances of successfully eradicating the introduced red fox population in Tasmania may be significantly higher than previously thought.

## Introduction

Understanding whether a species is extinct is important in a range of circumstances. For example in species conservation decisions need to be made about continuing funding and/or monitoring threatened species [1]. In eradication campaigns for invasive species decisions need to be made to cease or continue eradication programs [2]. In all cases this information can be used to reprioritise scarce resources. There are a range of data sources that can support analysis of extinction probability. Information that indicates presence of an organism is obviously relevant to this analysis. This includes data such as visual sighting, scats, hairs, tracks and sounds. The analysis will also typically depend on beliefs and understandings about the biology of the species and other key processes interacting with it. Ignoring these can lead to significant errors in inference [3].

A range of models exist that use sighting data to infer the likelihood of extinction, or to test the null hypothesis that a species is extant [4]. These existing analyses are closely related in that they share similar assumptions, in particular that there is a constant or declining sighting rate (equivalent to a constant or declining population). Whilst potentially reasonable for critically endangered species teetering on the edge of extinction, this assumption is unlikely to be realistic when dealing with invasive species with potentially robust demographics.

Invasion processes, as biological processes, are often complex in space and time and involve considerable stochasticity and uncertainty, hence stochastic simulation is a useful alternate starting point for exploration. For example, Wilson et al. [5] use a stochastic simulation approach to identify plausible scenarios for the establishment of invasive Burmese pythons (*Python molurus* Linnaeus 1758) in southern Florida. Their approach, however, stops short of using the data to undertake formal statistical inference about model parameters. The ability to infer properties of the model from data is crucial to logically align our observation of the world with the structure of our models.

Bayesian analysis is a well established approach to performing inference. A Bayesian analysis requires a prior distribution for parameters and a likelihood for the parameters given the data. Construction of appropriate likelihoods for sighing data is complicated. The likelihood of the observed data depends on the spatial arrangement of suitable habitats and the observation and demographic processes. Enumerating the possible process pathways that lead to particular observed data and deriving the associated likelihood terms is challenging in all but simple cases. For example Catteral et al. [6] consider modelling invasion dynamics of plants but assume that distribution data is observed without error at a number of time points. Pagel and Schurr [7] consider a very general framework for modelling species distribution data but only apply it to simple simulated data. O’Hara et al. [8] suggested using data augmentation techniques to accommodate missing data in simple occupancy studies, but the construction of these schemes so they are both computationally efficient and mix well is challenging [9].

The analysis of invasion data is more complex again. There can be uncertainty about the timing of the initial invasion and the state space of the model can be extensive. The complexity of realistic models in this case makes explicit probability (likelihood) calculations at best unattractive and at worse not feasible, as suggested by Marion et al. [10].

Approximate Bayesian Computational (ABC) methods provide a possible solution to analysing these complex problems. ABC methods allow inference for problems where it is possible to simulate data from the assumed model [11]. ABC methods are useful for inferring posterior distributions where likelihood functions are computationally intractable or too costly to evaluate [12]. This makes them well suited for making inference from stochastic simulation models [13], and they have been proposed as having application to making inference during biological invasions [14].

Here, using the apparent red fox (*Vulpes vulpes*, Linnaeus 1758) incursion into Tasmania (an island state of Australia) as an example, we illustrate the use of ABC methods for making inference on the demography, distribution and time of introduction of an invasive species, based on a realistic model of the invasion process combined with the data on the fox carcass discoveries by citizens arising from animal-vehicle collisions and hunter kills. Our results allow us to explore whether it is possible to reconcile the inference [15] of a widespread fox population (based on a range of evidence but heavily dependent on the extraction of fox DNA from predator scats), with that obtained by heuristic analysis of the temporal and spatial distribution of the fox carcasses discovered to date.

## Materials and Methods

### Tasmanian fox data

#### Tasmanian fox incursion

There is understandably much concern about the possible biodiversity impacts of an established red fox population in Tasmania. Although there have been several attempted introductions of foxes into Tasmania over the previous 100 years, there has never been any conclusive evidence of an extant population [16]. However, sometime between 1998–2001 an unknown number of foxes were purportedly introduced into Tasmania and shortly thereafter, the first of a number of fox carcasses were discovered around the central north coast and midlands areas [15,17]. Subsequently, the scat-derived DNA evidence has been used to infer that the population was widespread in northern, eastern and southern Tasmania as of 2010 [15]. We note that the evidence underpinning this inference is contested [18].

#### Carcass discovery data

Data on the discovery of fox carcasses were taken from publicly available data provided by the Invasive Species Branch of the Tasmanian Department of Primary Industries, Parks, Water and Environment (DPIPWE) (http://www.dpiw.tas.gov.au/). These carcasses are considered credible by authorities, comprising one hunter-killed fox in 2001 and three road-killed foxes (the last of which was discovered and reported in 2006) (Table 1). To avoid confusion we note that the relevant information is not limited to these four carcases. The failure to observe carcasses across extensive areas of Tasmania is also informative. It is this information (that no carcasses have been observed since 2006) that is the key observation underlying speculation about the status of the fox population in Tasmania.

**Table 1:** Details of fox carcasses found in Tasmania.

**Year**	**Location**	**Type**
2001	Symons Plains—the “Bosworth fox”	Hunter kill
2003	Burnie—“Burnie road kill”	Road kill
2005*	Lillico Beach, Devonport	Road kill
2006	Cleveland	Road kill

*Although officially reported as February 2006, this carcass was first sighted in December 2005.

### Approximate Bayesian Computation

#### ABC computations

ABC methods exploit the computational efficiency of modern simulation techniques by replacing the calculation of the likelihood with a comparison between the observed (*D*) and simulated (*D*’) data based on a model *[D|θ]* and a set of parameters *θ* that have prior distributions *π*(*θ*). The technical basis of the methods are explained in detail elsewhere, such as [12].

We construct a hierarchical model to describe the observed data. We create a simulation model of the foxes demographics and dispersal and incorporate uncertainties in our understanding of this using prior distributions on parameters. We then overlay a stochastic observation model to characterise the observation process, again reflecting uncertainties through prior distributions. The overall prior is *π*(*θ*).

The simplest ABC algorithm (ABC rejection sampler) in our case would then involve: (1) Sampling a set of model parameters θ* from the prior distributions *π*(*θ*) specified above; (2) Simulating a dataset *D*′ of road-killed and hunter-killed foxes using the hierarchical model; (3). Accepting the parameters θ* if a measure of the distance between the observed and simulated data is less than some arbitrary tolerance value *ε*(i.e. *d*(*D,D′*) ≤ *ε* where *d* is some distance function); (4) Repeating step one until a sufficient number of parameter combinations are accepted to characterise the posterior distributions of the parameters of interest. Whether or not the posterior distributions are correctly characterized depends on the tolerance between the observed data and accepted simulations. As *ε*→ 0, the correct posteriors are found with near certainty. The problem, of course, is that the probability of exactly reproducing the observed data may be so low that generating a sufficient number of samples for the posteriors may be computationally prohibitive. This is especially the case using rejection sampling which is inefficient due to repeatedly sampling areas of the parameter space that have a low probability of generating the observed data. Efforts to speed up the process generally involve the use of statistically sufficient summary statistics (if they exist) as a basis for estimating the discrepancy between *D* and *D*’, and the implementation of more efficient samplers. Toni *et al*. [12,19] provide more details.

Our measure of discrepancy was the sum over the observation period (conditional on the time of introduction) of the absolute difference between the number of observed and simulated carcasses, calculated separately for road kills and hunter kills. Approximate posterior distributions were generated using a an ABC sequential Monte Carlo (ABC SMC) sampler [12]— a particle filter. Starting with 1,000 particles (parameter combinations), the allowable discrepancy over the observation period between the simulated and observed road kill data was set to 2, 1, then finally zero. The allowable discrepancy for the hunter-killed data was set to 0 from the start. Hence for the final selection of accepted particles (and hence posterior distributions of parameters), the acceptance criteria was that the simulated road kills were exactly the same in number (*n* = 3) and timing (years 2003, 2005 and 2006) as was the simulated hunter kill (*n* = 1, year = 2001). No restriction was put on the location of simulated carcass discoveries, although this is clearly influenced by the value of model parameters. Thus there is additional information in the data that is not being used in the analysis. The reason for this is that the rejection rate of the sampler becomes too high if we incorporate these additional dimensions. Given this, the analysis is still well conditioned. The observed carcass discoveries are legitimate observation and the analysis conditions on this. While this data is aspatial, the interaction between the spatial observation and demographic processes means that a significant spatial component remains in the analysis.

Normal distributions were used as perturbation kernels for all parameters. We confirmed that the ABC SMC sampler was generating very similar posterior distributions to an ABC rejection sampler—our choice of the SMC sampler was to speed up computations.

### Spatio-temporal model of the invasion process

#### Purpose & type

In order to make inference about the likely demography, distribution and status of an invading fox population based on carcass discoveries, we require a spatio-temporal model containing the processes of interest. The purpose of the population spread model is to underpin inference on the spatio-temporal distribution of foxes in Tasmania, both now and into the future. To do this, the model needs to be simple enough that it is tractable computationally, and must marginalize to the process under question—are there foxes and where are they? In summary, the model must be “adequate” or “fit for purpose”.

We use a spatial model defined over a grid with each cell representing a 5 km × 5 km area which is deemed to be suitable or not to be colonized by a breeding fox population. This size cell is reasonable in terms of the typical home range size of a fox. Clearly this is a major abstraction of the fox population, but we argue it is adequate within the modelling framework used.

Assumptions include:

1. Introduced foxes are effective reproductive units from the first year of introduction (e.g. a least one male-female pair)
2. A female fox will always find a mate in each year (i.e. once occupied, a cell is capable of reproducing in each year).
3. The number of road kills in a cell is trivial in terms of cell population dynamics (i.e. has no effect on cell reproductive performance)
4. The hazard of being hit on a road is constant for all major roads.

Clearly some of these assumptions can be removed or relaxed (e.g. getting traffic volume data to underpin the hazard rate on roads). The ABC method requires that estimable parameters are assigned prior distributions. The details of these priors are given below.

#### Habitat suitability

The prior distributions of habitat suitability are based on previous empirical studies conducted in mainland Australia summarised in [20]. The following land use classifications were deemed suitable to sustain a fox population—grazing of native pastures, forestry, plantations, modified pastures, cropping, horticulture, irrigated pastures and cropping, irrigated horticulture, intensive animal and plant production, rural residential, urban intensive uses and land in transition. Land use classifications deemed unsuitable were nature conservation (predominantly south-west Tasmania), managed resource protected areas, other minimal uses, and mining and waste. This produced a fox habitat suitability map with 48% of Tasmania deemed suitable for foxes, and a spatial distribution very similar to that of [17] and [15].

#### Locations of purported introductions

Introductions were assumed to result in a breeding pair of foxes which are assumed to form a functional “occupied” cell. Based solely on the rumoured/alleged release locations of foxes [17], in each simulation we introduced foxes to cells near Longford, Oatlands, and St Helens. Clearly the distance from a major road that the introduced population first establishes could have a large effect of the timing of the observed road kills. To account for this, for any one simulation, first, the distance from a major road of the introduction was drawn from a uniform prior distribution on 0 to 25 km. An introduction cell with this setback from a major road was then selected with equal probability from the set of grid cells located within 30 km of the townships at the centre of the rumoured release locations. Note that we do not include the possibility of self- introduction at a major port, for which one case has been documented, and could have been occurring for many decades.

#### Timing of introductions

The year of first introduction is rumoured to be 2001, although “accumulated evidence also indicates that such an act may have also occurred in 1999 and 2000” [17]. We backdate the rumoured date to include the possibility that foxes were introduced as early as 1995, and use a uniformly distributed prior on the period 1995–2001.

#### Dispersal

Two dispersal kernels were used, one to reflect what could be considered “natural” dispersal behaviour within an established fox population, and the second “invasion” dispersal behaviour to accommodate the hypothesis (untested) that the invading low density (and persecuted) fox population could have different dispersal characteristics due to the lack of con-specifics. The natural spread kernel is based on a Weibull distribution fitted to the data of Coman *et al*. [21], who found that the great majority (70%) of red fox cubs were recaptured within 2 km of their original tagging location. For those that dispersed further, dispersal distances ranged up to 30 km with a mean of 11 km. The invasion kernel is circular, though with a flat density out to 30 km in all directions surrounding the occupied cell.

#### Survival & reproduction

The cell survival parameter (*q*) is the probability that an occupied cell will retain a breeding fox population from year-to-year. A uninformative prior (Uniform [0,1]) was chosen, reflecting genuine uncertainty as to the effectiveness of the fox eradication program (e.g. no confirmed bait take by a fox as of end 2013), and the behaviour of the invading fox population, and the fact that past introductions have apparently failed [16]. The cell reproduction number (*λ*) which is the number of new occupied cells generated by occupied cell is similarly uncertain. Predation by Tasmanian devils (*Sarcophilus harrisii*, Boitard 1841) on fox cubs has been hypothesized anecdotally as a reason for failure of past introductions of foxes, though with the spread of devil facial tumour disease (DFTD) since about 1995 and associated major decline in devil abundance across much of northern and eastern Tasmania by 2006 [22], this hypothesized effect may be weakened considerably. A uninformative prior (Uniform [0,2]) was used for *λ* (i.e. somewhere between zero and the expected number of female foxes per litter assuming a 50:50 sex ratio). The net reproduction rate (*NRR*) for this simple system assuming survival before reproduction is (using the formula for the sum of a Geometric series):

NRR=λq+λq2+λq3+...=∑n=0∞λqn−λ=λ1−q−λ

A *NRR* above unity is a necessary (though not sufficient) requirement for a population to establish.

#### Probability of road-kill

Roads are a strong attractant for scavenging species such as foxes and they are at substantial risk of being involved in collisions with vehicles. For example, Snow *et al.* [23] estimated the annual survival rate for San Clemente island foxes (*Urocyon littoralis*, Baird 1857) was about 20% lower for individuals living near roads. We used the record of fox control on Philip Island [24] to help inform a prior on the probability that an occupied cell with a road passing through it will generate a road kill in any year. Over the 25-year period 1979/90–2004/05, there were 1,000 foxes recorded as being removed from Phillip Island, of which 35 were road kills. The fox population was thought to number at least 120–140 (removals of known cohort members) from 96/97 to 99/00. The island is about 100 km^2^, which would be the equivalent of four 5 km × 5 km square cells, which could reasonably be assumed occupied in each of the years. So, the rate of road kill is 0.35 cell^−1^ year^−1^. The corresponding probability of at least one road kill will be about 0.30 cell^−1^ year^−1^. The population density of foxes in Tasmania is postulated as lower than that on Phillip Island, which also has a much higher density of roads, so the 0.30 was considered an upper bound. We were conservative on the low side, and used a Beta (10,90) distribution which has a mean of 10%, though considerable probability mass between 5% and 15%.

#### Probability of shot-kill

The hunting “observational” process in Tasmania is substantial, with in excess of 4,000 deer hunting licenses issued annually by DPIPWE to hunt the fallow deer (*Dama dama*, Linnaeus 1758), whose range overlaps extensively with the habitat considered suitable for foxes, though we note a lack of overlap on the northern coastal fringe. Elsewhere hunting of small introduced game species (e.g. European rabbits *Oryctolagus cuniculus* Linnaeus 1758, European hares *Lepus europaeus* Pallas 1778) is a popular pastime across Tasmania (author’s personal observation). In addition, permits are issued for the control of native species that can cause browsing damage to agriculture, such as wallabies (*Macropus spp.*) and brushtail possums (*Trichosurus vulpecula* Kerr 1792). For the 2012–2013 season Wallaby licenses alone numbered 7,236 (DPIPWE – February 2013). Spotlight shooting, which is a recognized method to obtain fox carcasses, is a core method for much of this hunting. This effectively extends the hunting observational process to all of Tasmania other than the nature conservation estate. Despite the widespread nature of hunting in Tasmania, its effort is undoubtedly uneven in time and space. To allow for this, at the start of each simulation run, the probability of a cell being subject to potential hunting was randomly assigned using a 50% probability—that is hunting could occur on half the cells. This is an arbitrary choice on our part, and its main role is to accommodate for the possibility that a fox population is extant in a cell not subject to “observation” by shooting.

Generating a prior on the probability of shooting generating a shot fox from an occupied cell subject to hunting was difficult. Field *et al.* [25] estimated the probability of detecting foxes within a 1 km segment of a spotlight transect ranged between 6 and 18% depending on vegetation, but translating this to a typical annual probability of successfully shooting a fox within a 5 km × 5 km cell is difficult. We could again use the Phillip island data, although the intensity of spotlight searching effort was very high there—possibly much higher than would be typical for a hunted area in Tasmania. In the end we settled on a Beta (10,90) distribution (same as the probability of a road kill). This is possibly conservative on the low side, as Heydon & Reynolds [26] report a culling rate from Britain that would correspond to a much higher probability.

#### Ordering of events

The ordering of events applied to occupied cells were survival, road or hunter kill, then reproduction.

#### Estimating time to next carcass discovery or extinction

The trajectories for accepted simulations (see below) with an extant fox population as of the end of 2013 were projected stochastically out to 2022 using the parameters and state variables (e.g. spatial location of occupied cells as of 2013) unique to that simulation. These projections were used to estimate the time to the next fox carcass being detected, or the population going extinct, or either of these outcomes occurring.

#### Implicit prior on extinction probability and population size

The choice of priors corresponded to an implicit prior probability of population extinction at the end of 2013 of 39.1%. Conditional on the population being extant, the number of occupied cells was variable (95% C.I. = 2–933) though typically large (median = 191).

### Model assessment

#### Exploring prior sensitivity

The sensitivity of the results to the choice of prior distributions may be of interest. Under the Bayesian paradigm if priors are formulated consistently based on one’s beliefs there should not necessarily be a requirement to present results arising from different priors. We note, however, that different people may have different prior beliefs, and hence a sensitivity analysis will assist their interpretation of the results. To facilitate this we conducted an analysis to investigate the sensitivity of our results to reasonable modifications of the prior distributions. The approach of rerunning the ABC computations with different priors is difficult to implement for our model due to the time needed to undertake computations. We can, however, use importance sampling to generate revised posterior distributions arising from alternative prior distributions. Briefly, using the modified prior distribution *π*′(*θ*) as the importance function we calculate the importance weights as *π*′(*θ*)/*π*(*θ*) and resample the posterior distribution with replacement using these weights to obtain the alternative posterior distribution[27].

The most informative prior parameters in our model relate to the observation process through road kills and hunting, hence it makes most sense to explore the sensitivity of the results to changes in prior distributions for these parameters. As the number of samples from our posterior distribution is reasonably small (n = 1,000), we are restricted somewhat in how far we can change the prior distribution before the importance weights become degenerate. We therefore explored increasing/decreasing the expected yearly probability that an occupied cell traversed by road generates a roadkill by ± 10% using the appropriate Beta distributions. Likewise, we explored the impact of altering the expected yearly probability that an occupied cell subject to hunting generates a hunter kill by ± 10%.

#### Model Fit

An important requirement of any model is that its implicit properties are consistent with the observed data. To explore this we compared the observed data with the predictive distribution. Samples from the predictive distribution were generated by sampling parameters from the posterior and then using the simulation model to generate observations. We also simulated the prior predictive distribution by sampling directly from the priors. This allowed us to explore the influence of the priors.

#### Software & hardware

All calculations was undertaken within the R computing environment [28] using the “raster” [29] and “simecol” [30] packages. Ten computer processor cores were utilised to run ten independent ABC SMC simulations with the random number generator for each processor seeded with a different starting value using the set.seed() function within R. Approximately 5,000 hours of processor time were required – considerable time savings would be expected if programmed more efficiently and/or in a faster computing environment.

The source R code is available on request from the corresponding author.

## Results

### Population parameterization underlying accepted trajectories

**Figure 1:**
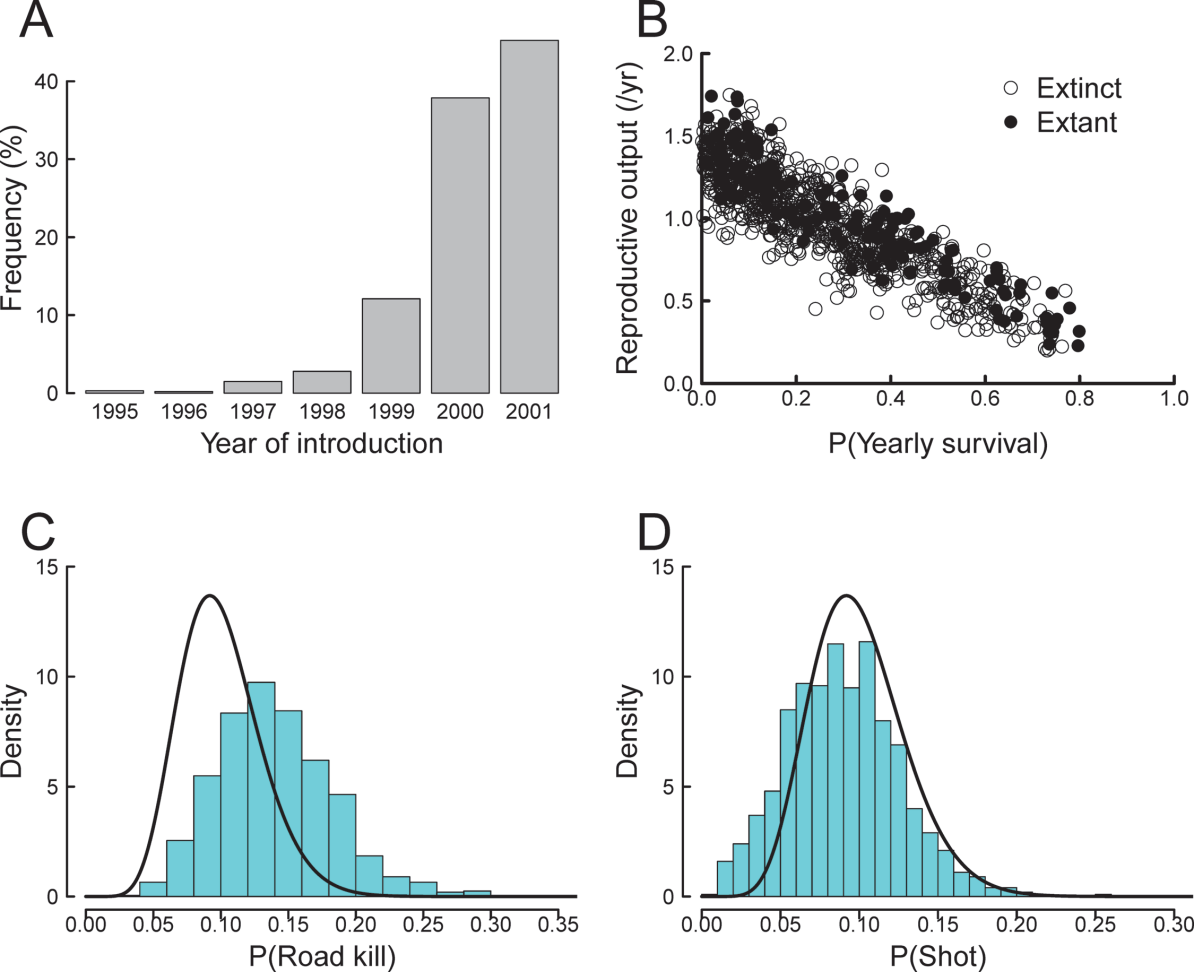
Approximate posterior distributions for: (A) Year of introduction; (B) Relationship between reproductive output per year and probability of yearly survival, with open circles indicating populations extinct as of end 2013; (C) The probability density of being a road kill (bars) and the prior based on a Beta distribution (solid line); (D) The probability density (given hunting occurs in an occupied cell) of generating a hunter kill (bars) and the prior based on a Beta distribution (solid line).

The most likely year of introduction was strongly skewed towards either 2001 (45% probability) or 2000 (38% probability), with the year 1999 somewhat plausible (12% probability) and earlier years unlikely (Fig. 1A). The approximate marginal posterior distribution of the distance from the introduction site to a major road showed weak though consistent trends, with a slight preponderance for the releases being 5–10 km from a major road as opposed to either nearer or further. There was no correlation between the release distances (e.g. a distance release at one location is correlated with a closer release elsewhere).

The parameter space of accepted simulations shows an unsurprising strong dependence between the yearly cell reproduction number and yearly survival (Fig. 1B). The estimated annual probability of a road kill in an occupied cell traversed by a road is significantly changed from the prior (Fig. 1C), with a modal value of about 13%. Higher road kill probabilities (e.g. >20%) are considered plausible, and lower probabilities less probable (Fig. 1C). At first glance, the estimated annual cell-based probability of a hunter kill in cells subject to hunting is only slightly changed from the prior (Fig. 1D), however closer inspection reveals a considerable increase in probability in the lower tail of the distribution. That is, despite the prior entertaining little chance of the hunting risk being very low (e.g. 1–2%), the analysis suggests this as a possible solution to the observed data. There was negligible difference in the inferred mode of dispersal (“invasion” 53% vs. “natural” 47%), hence we have no support for discarding either.

**Figure 2:**
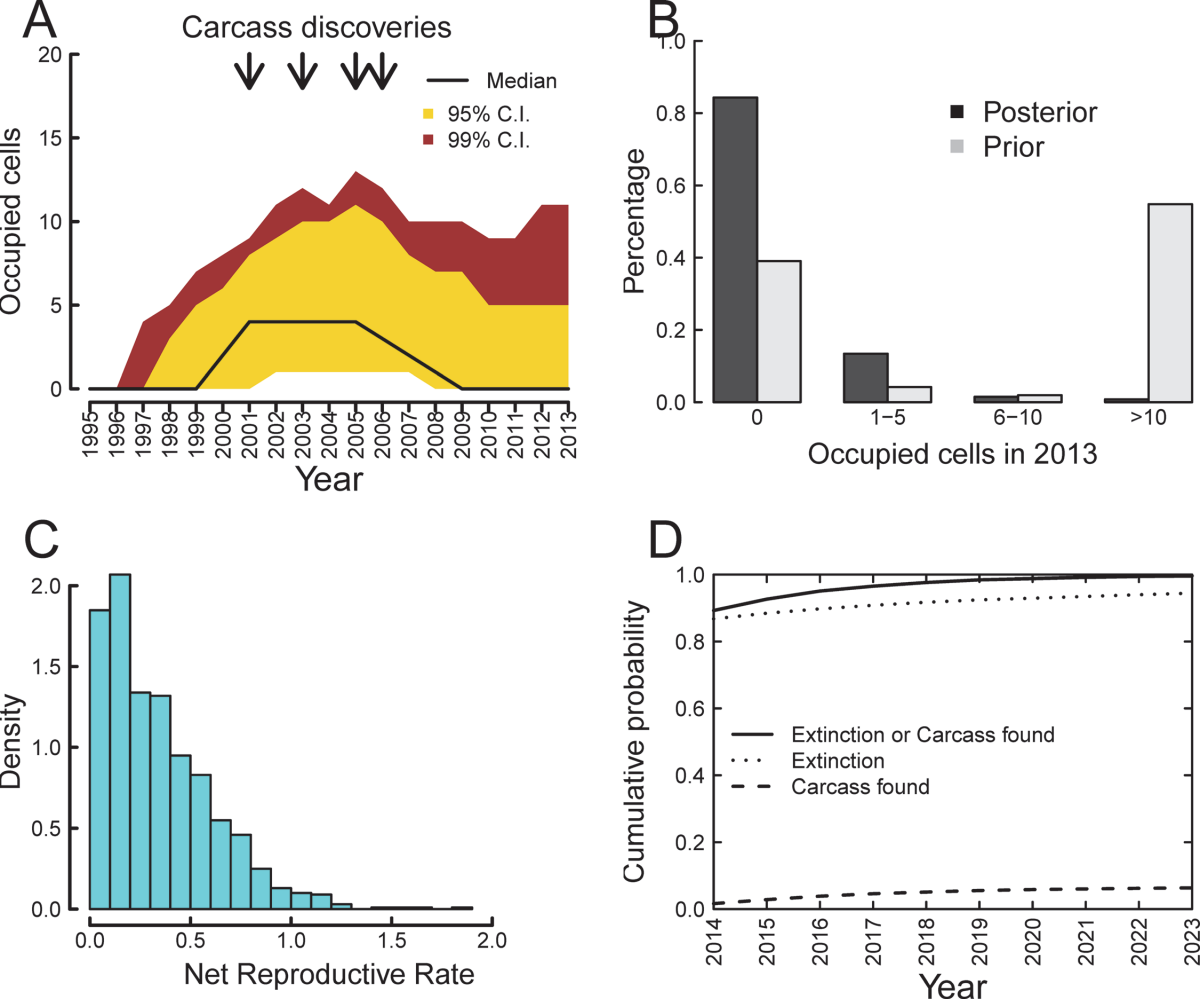
From 1,000 accepted simulation runs exactly matching the observed number of carcass discoveries and hunter kills in time and number as of end 2013. (A) Median (solid line) population trajectories with 95% credibility (gold) and 99% credibility intervals (brown). Arrows indicate years of carcass discoveries; (B) Frequency distribution for the number of occupied cells at the end of 2013 out of 1256 habitable cells. Solid bars are posterior, open bars correspond to the prior; (C) Approximate posterior distribution of the Net Reproductive Rate (*NRR*) of occupied cells. (D) Cumulative probability of population extinction (dotted line), cumulative probability of at least one new road kill (dashed line), and the cumulative combined probability of either population extinction or at least one further road kill (solid line). Results are based on 30 simulation runs on each of the 1,000 accepted runs.

The number of occupied cells for trajectories of accepted simulation runs are broadly similar. They are small populations with low reproductive rates at substantial risk of extinction, although for a small proportion of accepted simulations the population extant as of end 2013 was more widespread (Fig. 2A). 50% of accepted trajectories had become extinct by 2009.

The inferred population as of the end of 2013 is most likely extinct (*c*. 84% probability), or highly restricted (1–5 occupied cells) with about 13% probability. There is about 2% chance the population occupies either a small (6–10) or moderate–large (>10) number of cells (Fig. 2B). To put this in context, if the population is still extant then it is mostly likely has a distribution of a similar size to the initial release. This posterior inference is dramatically different from that implicit in the chosen priors, where a moderate-large number of occupied cells was the most likely outcome (Fig. 2B). The information contained in the observation processes (hunting, vehicle collisions) clearly heavily discounts against such a large final population.

The net reproduction rate suggests the population most likely has low demographic vigour, with only 2.6% of accepted models having a life time reproductive rate above one (Fig. 2C). This infers the long-run minimum probability of extinction, ignoring stochastic misfortune, is 97.4%. For the 13% of accepted population simulations that were still extant as of end 2013, the conditional probability of the net reproductive rate being above one was higher (7%).

### Estimated time to next detection or extinction

Conditional on the model assumptions and prior beliefs, the probability that a further carcass is found in 2014 is estimated to be about 2.5% and the probability that the population has become extinct increases to *c*. 87% (Fig. 2D). Extending this into the future, by 2023, it is estimated that there is nearly 100% certainty that either the population has become extinct, or it has been detected by either a road or hunter kill (and hence it is apparent that further control is necessary) (Fig. 2D). That is, within 10 years, either extinction will be achieved with probability of about 95%, or it will be apparent that attempts to cause population extinction have failed. This inference, as previously stated, is conditional on a model fitted to road-kill and hunter-kill data only.

**Figure 3:**
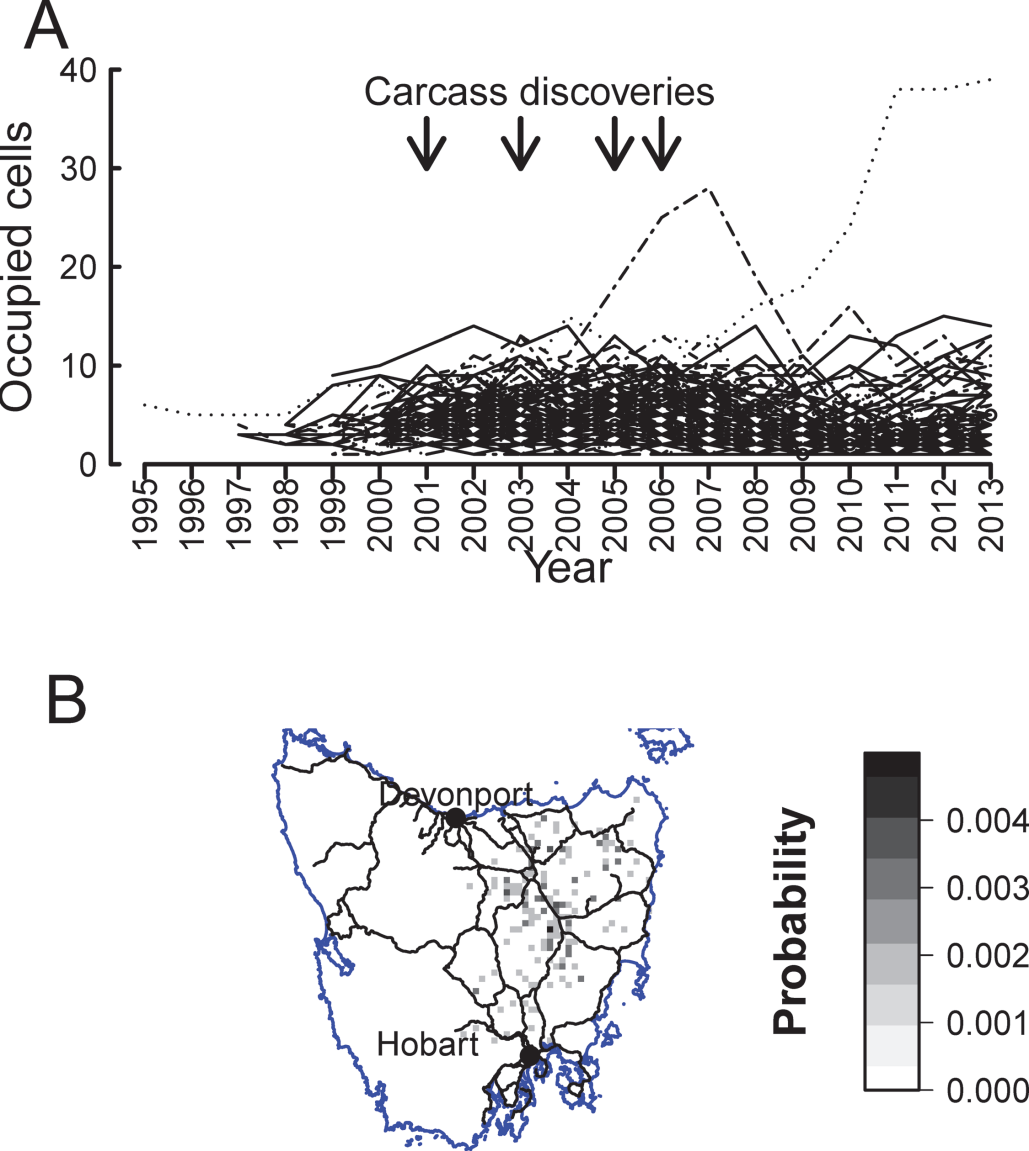
Results from approximate posterior distributions. (A) Trajectories of accepted simulation runs for which the population was extant as of the end 2013. (B) Approximate posterior distribution of the distribution of cells occupied by the red fox in Tasmania as of the end 2013. Solid lines are major roads.

If the population is not extinct as of 2013 (i.e. conditioning the results on those extant as of end 2013), the median number of occupied cells was 2 (one-sided upper 95% credibility interval 9 cells). To put this in context, this is slightly less than the assumed number of occupied cells arising from the original release. Note, however, the low possibility (in this case 1 in 1,000) of a quite widespread population occupying 39 cells as of end 2013 (Fig. 3A).

Regarding the spatial distribution of the population, the posterior distribution of cell occupation at the end of 2013 is characterised by very low occupancy probabilities (Fig. 3B). Cells with higher probabilities of occupancy tended to be located away from major roads (Fig. 3B). The posterior 2013 distribution of foxes inferred by our ABC results is not consistent with the widespread distribution inferred by [15].

### Exploring prior sensitivity and model fit

The estimated probability of extinction as of end 2013 is moderately insensitive to small increases in the prior probability of a cell generating a road kill or a hunter kill, either singly or in combination (Table 2). The probability of extinction was more sensitive to a decrease in the prior probability for hunting, with a 10% reduction in the hunting probability resulting in a decrease to the probability of extinction by about 5%. If a 10% decrease in the road kill probability was also included, then the probability of extinction decreased by a total of 8.6% (Table 2).

**Table 2:** Sensitivity of extinction probability as of end 2013 to changes in the mean expectation of roadkill and hunting parameters.

***P*(Roadkill)**	***P*(Hunter kill)**	***P*(Extinction)**
0.11 (+10%)	0.10 (n.c.)	0.857 (+1.7%)
0.09 (−10%)	0.10 (n.c.)	0.823 (−2.4%)
0.10 (n.c.)	+10%	0.861 (+2.0%)
0.10 (n.c.)	−10%	0.803 (−4.8%)
0.11 (+10%)	0.11 (+10%)	0.872 (+3.5%)
0.09 (−10%)	0.09 (−10%)	0.771 (−8.6%)

Values in parentheses represent percentage changes from either model parameter expectation of posterior model result. “n.c.” indicates no change.

**Figure 4:**
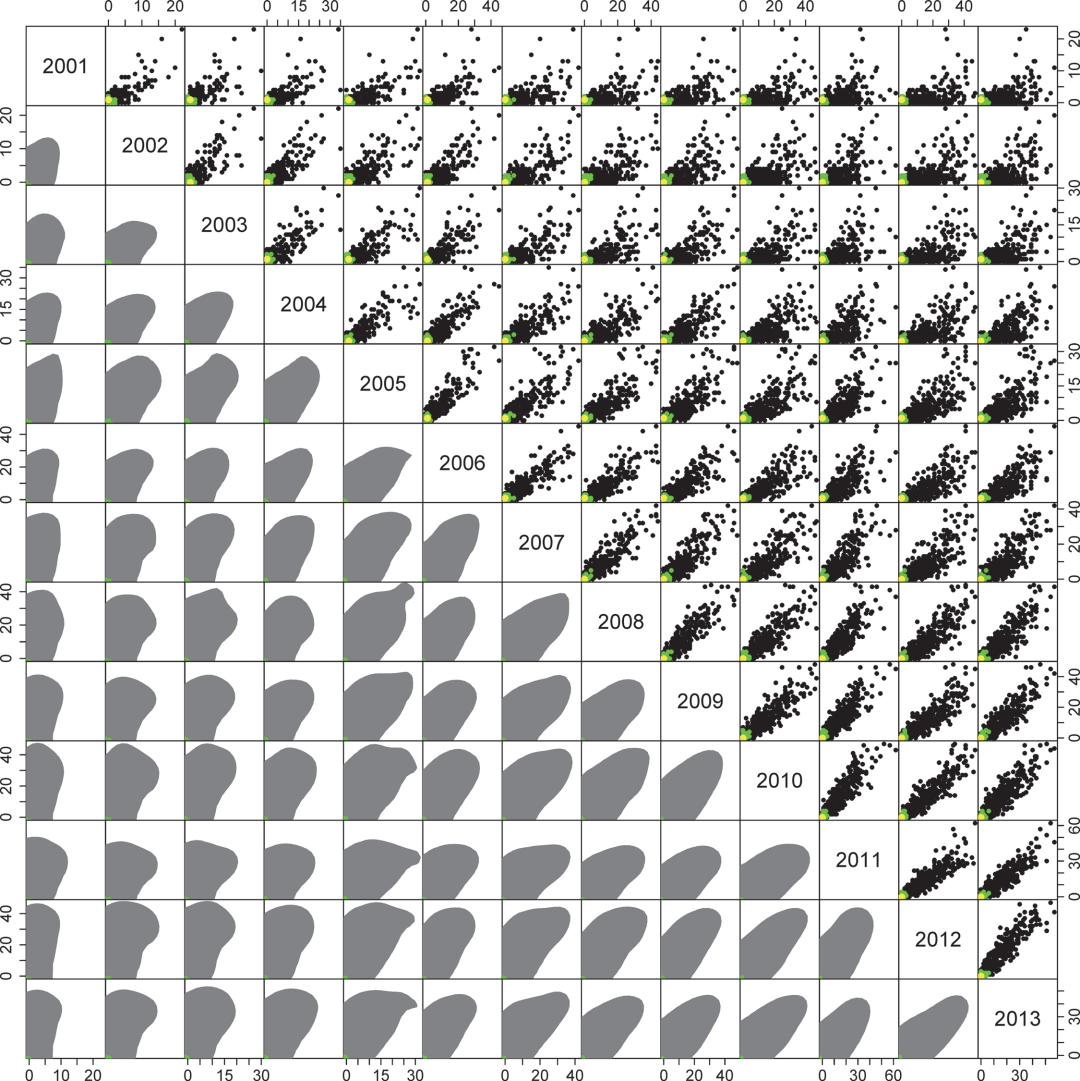
Comparison of yearly carcass discoveries for prior and posterior distributions, and the observed data. Data are 1,000 draws from the prior (black points) and posterior (green points) distributions, and observed data (yellow point). Lower diagonal illustrates 99% highest density regions for prior (grey shading) and posterior (green shading) distributions.

The predictive distributions of carcass discoveries under the prior and posterior, along with the observed data, are displayed in Fig. 4. From this plot we note the significant impact of the data on the priors and the consistency of the observed data with the predicted values based on the prior distribution. Thus we can conclude that our data is not inconsistent with the assumed model.

## Discussion and Conclusions

Our inference of the Tasmanian fox population based on our analysis most strongly supports an extinct population as of the end of 2013, or a small population with low population vigour. A more widespread and possibly increasing population is inferred to be possible though with low probability. This inference is not consistent with the inference of [15] based on extracting fox DNA from predator scats and confirms the initial heuristic reckoning that the two forms of data produce substantially different inference. Indeed, on the basis of both sets of publicly available data, citizens have reported that the inferred widespread fox population doesn’t make sense to them, either directly through numerous online postings (e.g. find examples by searching for “fox” at the Tasmanian Times http://tasmaniantimes.com), or in responses to surveys [31].

We note that applying our methods to the scat DNA data assuming 100% test specificity and ignoring the carcass data will generate inference little different to that of [15] – a widespread fox population. We note, however, that the inferred parameters for the probability of foxes being involved in collisions with vehicles or shot by hunters will likely be so low as to be implausible. Analysing the two data sets is problematic assuming 100% specificity. A preliminary analysis suggests that the combined data are inconsistent with the process models considered here. The discrepancy between our inference based on carcass discoveries and that based on the extraction of fox DNA from predator scats is worthy of further quantitative investigation.

There is, however, no reason for our inferences based only on carcass data to not be considered as independent and valid – they are based on modelling principled modelling process that is informed by reasonable prior beliefs and properly conditioned on the data. Our results align with some people’s heuristically-derived low belief in a widespread and spreading fox population in Tasmania, in contrast with published inference [15]. Demonstration of a logical basis for these beliefs is important. It has been argued that the persistence of widely held mental models that reject the incongruent ‘stimulus’ of a widespread fox population is reason to consider alternative communication strategies to directly address altering such mental models [31]. Our results suggest the mental model may not be the one in need of alteration.

There are several possible explanations for the discrepancy between this analysis and [15]. Under the hypothesis that foxes in Tasmania are widespread, the explanation given for the lack of hunter kills (despite Tasmania having a large and active hunting fraternity spread across the state), or verifiable sighting data (e.g. photographic evidence) is that individual foxes are elusive and cryptic [15,17] making it possible that a low density population would remained undetected by these means. Whether this remains true for a population of individuals clearly depends on the size of the population and its spatial and temporal overlap with observation processes. Our approach to the analysis allows such explanations of low detectability to be quantitatively evaluated in light of the observed data. We accept our representation of the hunting observation process is an approximation of what occurs in reality. It is possible that peri-urban areas, particularly in the north of the state may have lowered detection levels due to lowered levels of hunting. Our model formulation does allow some areas to have zero detection probability, but may not be elaborate enough to account for such specific possibilities.

Why the population reproductive rate of an introduced fox population would be so low in Tasmania with its varied and abundant small mammal population (including the European rabbit– a known dietary staple of the fox) is a puzzle, unless the founding population (indeed rumoured to be small) succumbed to stochastic misfortune, or possessed low vigour. It would not be without precedent; previous introductions of foxes to Tasmania have failed [16], and at least two introductions of foxes to the Isle of Man ultimately failed, despite the population establishing and numbering in the 100s for the most recent introduction [32]. In contrast, the founding population of introduced foxes on the Australian mainland was also small, yet once established they had spread extensively and visibly over *c*. 13,000 km^2^ approximately a decade following their introduction [33]. In Tasmania, possibly the hypothesized (though untested) effect of Tasmanian devil predation on fox cubs is highly non-linear and still occurs at low devil population densities. Tasmanian devils still persist in low densities in the area where DFTD first emerged in the north-east of the state [22], as evidenced by camera trap data for these areas (Sam Thalmann unpublished data). These data from 30 camera traps spread in a grid over 3,000 km^2^ over the North-east tip of Tasmania identified 34 individual Tasmanian devils in a population that is considered sparse. No foxes were sighted, despite several fox DNA positive scats having been found in the general area [15], lending weight to inference that fox population is small if indeed it is extant in this region. We note there are additional camera trap data arising from the fox eradication program that could be used to further extend the inferences from the model to another form of potential sighting data. Any such additional observation processes that have not detected any foxes can only further limit the estimated distribution of the population.

Although the model can continue to be made more elaborate in many ways, these possible improvements need to be evaluated in the light of model purpose. As a means of making broad inferences on the distribution of foxes we consider it adequate. It represents the key processes that underpin the size and distribution of the fox population and the rate of carcass discovery. We do not believe that further elaboration of the model with secondary processes will significantly improve inferences, although how this is determined in particular cases will always involve significant amounts of judgement. An example of a more complex model elaboration is given by [34].

The analysis of the fox data has highlighted a number of issues with this type of analysis. First, the nature of the data means that there is some sensitivity of the results to the prior. Rather than being a weakness, this sensitivity reflects the importance of using a Bayesian analysis. Policy makers need answers to questions based on the available data and the analysis presented does this in a transparent way. The alternative approach of simply seeking an expert’s view arguably contains more prior opinion than the analysis we have presented. The sensitivity to priors underscores the “two-edged sword” of Bayesian inference—the ability to include prior beliefs is both a blessing and a curse as different individuals often have different prior beliefs leading to different inferences.

We note also that sensitivity to a prior is not the same as the data being uninformative. The observed data in this case effectively rules out a large number of possible processes and outcomes (see Fig. 2B). The issue of prior sensitivity means that it is important to justify priors and not rely on the usual uninformative defaults. It also suggests that alternative priors/sensitivity analysis be considered so that others involved in decision making process can explore their own beliefs.

The techniques we have presented here are potentially more widely applicable to analysing data on invasive organisms. Example where it would be well suited include inferring the possible distribution of an organism when it is first discovered and assessing the progress of a disease eradication program in a population. In these cases, the ability to incorporate expert opinion and potentially complex processes is a distinct advantage. There are, however, still significant challenges in this analysis that should not be understated. While the logic of the analysis is applicable to all processes and data, the computational efficiency of the algorithms are still low. More complex data will potentially further degrade the efficiency of the algorithms as the space to search becomes computationally daunting. Increased computing resources and development of more efficient algorithms [11] will improve this situation, but for the foreseeable future these techniques will be restricted to problems with low dimensional data.

A referee was interested in how these techniques could be validated. At one level this question is not well posed. The approach logically reconciles and updates beliefs and observed data. Interpreted in this way the technique is internally coherent. Viewed in a decision making context the question is better defined. Does this analysis provide a more accurate prediction of outcomes than experts forming views based on their knowledge and mental reckonings? Studies could be done to compare the two approaches.
